# Event-Triggered Adaptive Consensus Control for Nonlinear Multi-Agent Systems with Prescribed Performance and Full-State Constraints

**DOI:** 10.3390/s26154860

**Published:** 2026-08-02

**Authors:** Wenjie Wang, Qian Chen, Huiying Xu, Zhendong Chen, Longfei Wang, Deang Su, Xinzhong Zhu

**Affiliations:** 1 Shenzhen Robotmeta Technology Co., Ltd., Shenzhen 518103, China; 2 Zhejiang Key Laboratory of Intelligent Education Technology and Application, Zhejiang Normal University, Jinhua 321004, China; 3 College of Computer Science and Technology, Zhejiang Normal University, Jinhua 321004, China

**Keywords:** multi-agent systems, event-triggered control, prescribed performance control, full-state constraints, barrier Lyapunov function, adaptive neural control

## Abstract

This work addresses the output agreement problem for networked nonlinear agents with simultaneous transient performance specifications and state limitations. A performance function confines synchronization deviation of each agent within a user-designed envelope that quantifies both convergence speed and permissible steady-state offset. Logarithmic barrier certificates are embedded into the recursive control construction to prevent any state variable from exiting its admissible region. Unknown plant nonlinearities are compensated via Gaussian radial basis function approximators, and a first-order filter bypasses the repeated analytic differentiation that complicates traditional recursive designs. To reduce frequent actuator adjustments, each agent’s control signal is refreshed only at aperiodic instants governed by a local dynamic triggering rule depending exclusively on its own measurements. A Lyapunov analysis confirms that all closed-loop trajectories remain semi-globally uniformly bounded, the synchronization errors obey the imposed performance limits, the state restrictions are never breached, and infinitely many triggering attempts cannot accumulate in finite time. The theoretical findings are corroborated through a computational experiment involving four heterogeneous followers coordinated by a single virtual leader.

## 1. Introduction

The past twenty years have witnessed intense investigation into distributed coordination paradigms for multi-agent networks, fueled by their relevance to formation flying of satellites, autonomous drone swarms, smart traffic management, and distributed sensing [1,2,3,4,5,6]. The central coordination primitive is often termed consensus or synchronization and requires every participating node to align its state or output trajectory with those of its peers, relying exclusively on locally exchanged information [7,8,9].

Real-world deployment of agent teams invariably confronts two categories of restrictions: behavioral specifications and physical limitations [10,11]. Regarding the former, the quality of the transient response is measured through attributes such as percentage overshoot, settling speed, and residual tracking offset, which is usually as important as asymptotic stability. The prescribed performance control (PPC) philosophy, pioneered by Bechlioulis and Rovithakis [12], addresses this need by imposing a time-varying boundary on the tracking error and then applying an invertible mapping that transforms the constrained regulation task into an equivalent unconstrained one. This technique has been successfully ported to diverse nonlinear plants [13,14,15,16] and, more recently, to networked agent systems [17,18,19].

Concerning physical restrictions, operational safety and hardware limits dictate that system variables, such as joint positions, velocities, and voltages, must never leave designated safe regions [20,21]; otherwise, degradation or catastrophic failure may ensue. Barrier Lyapunov functions (BLFs), which diverge to infinity as their arguments approach prescribed bounds, have proven to be an especially effective instrument for enforcing such constraints within Lyapunov-based control designs [22,23,24]. Several research efforts have sought to marry PPC with BLF techniques to concurrently meet transient specifications and state confinement requirements [25,26].

A separate but equally important concern in digitally implemented control architectures is the frugal use of communication and computation resources. Periodic sampling strategies, though simple to analyze, often waste bandwidth and processing power by executing updates even when the system is quiescent. Event-triggered control (ETC) offers a more economical alternative: the actuator signal is recomputed and transmitted only when a state-dependent criterion signals that a refresh is necessary [27,28,29]. Migration of ETC ideas to the multi-agent domain has yielded a rich body of results [30,31]. Among the various ETC paradigms, dynamic triggering rules that augment decision logic with an auxiliary internal state can deliver noticeably sparser update sequences than their purely static relatives [32,33,34,35].

Notwithstanding the individual maturity of PPC, BLF, and ETC research streams, their simultaneous integration for nonlinear agent networks has not been satisfactorily accomplished. Published contributions typically tackle at most two of the three facets. Zhang et al. [18] combined PPC with fuzzy ETC but omitted state constraints. Similarly, Chang et al. [36] investigated event-based adaptive formation and tracking with predetermined performance, further demonstrating the viability of combining ETC with performance guarantees. Long et al. [25] delivered fixed-time coordination with prescribed accuracy and state bounds but assumed continuous controller execution. Zong et al. [26] considered state-delayed agents with constraints and event-based updates yet did not incorporate performance envelopes. The present article is therefore directed at closing this gap: devising a single control architecture that unifies prescribed output behavior, hard state bounds, and dynamic event-driven actuation for nonlinear strict-feedback agent dynamics.

In light of the preceding discussion, the novel contributions offered by this manuscript are:

1. An integrated control strategy is synthesized for the first time in the paper, which simultaneously delivers prescribed transient/steady-state accuracy guarantees, respect for hard state bounds, and event-based control execution for nonlinear strict-feedback agent teams.
2. The proposed design fuses the PPC error remapping, logarithmic barrier certificates, and radial basis function approximators within a single recursive backstepping procedure incorporating dynamic surface filtering, thereby ensuring that synchronization deviations honor the specified envelopes while every state variable respects its confinement limits.
3. A distributed dynamic event-triggering law is formulated whose parameters can be tuned to regulate the trade-off between update sparsity and regulation fidelity; it is formally established that the triggering sequence cannot exhibit an accumulation of events in any bounded time window.
4. The computational tractability of the scheme is enhanced by employing neural approximators to handle unknown agent nonlinearities and by invoking first-order command filters to eliminate the algebraic complexity that plagues conventional backstepping implementations.

The exposition proceeds as follows. Section 2 collects the necessary mathematical background (graphs, agent models, performance functions, barrier certificates, and neural approximators) and states the control objectives. Section 3 details the recursive construction of the event-driven adaptive regulator and provides the Lyapunov-based closed-loop analysis. Section 4 validates the theoretical developments through numerical experiments on a four-agent system. Concluding remarks and directions for ongoing investigation appear in Section 5.

## 2. Preliminaries and Problem Statement

### 2.1. Notations

Throughout the article, R stands for the real line and N for the set of nonnegative integers; $R^{n}$ is the standard *n*-dimensional vector space. The symbol ∥·∥ indicates the Euclidean length of a vector or the induced spectral norm of a matrix. $\mathrm{diag}\{a_{1},\ldots ,a_{m}\}$ constructs a diagonal array from the listed entries. A superscript ${\left(\cdot \right)}^{T}$ denotes transposition and $\lambda_{\max}\left(\cdot \right)$, $\lambda_{\min}\left(\cdot \right)$ return the largest and smallest eigenvalues, respectively.

### 2.2. Graph Theory

An agent network comprising *N* followers, indexed by 1 through *N*, and one leader designated as node 0 is considered. Information exchange among these N+1 entities is captured by a directed graph (digraph) $\bar{G}=\left(\bar{V},\bar{E}\right)$, whose vertex collection is $\bar{V}=\left\{0,1,\ldots ,N\right\}$ and whose arc set $\bar{E}\subseteq \bar{V}\times \bar{V}$ encodes the allowed communication directions. The ordered pair (j,i) belongs to $\bar{E}$ precisely when node *i* is capable of receiving data transmitted by node *j*. The adjacency structure is represented by $\bar{A}=\left[a_{ij}\right]\in R^{\left(N+1)\times (N+1\right)}$, whose entries satisfy $a_{ii}=0$, $a_{ij}=1$ for $\left(j,i\right)\in \bar{E}$, and $a_{ij}=0$ otherwise.

For the purpose of distinguishing those followers that can directly sense the leader, the diagonal indicator matrix $B=\mathrm{diag}\{b_{1},\ldots ,b_{N}\}$ is introduced with $b_{i}=a_{i0}$; thus $b_{i}=1$ if follower *i* has access to the leader signal and $b_{i}=0$ otherwise. Let $L_{f}$ denote the Laplacian of the subgraph restricted to the *N* followers. The composite matrix $H=L_{f}+B$ then encapsulates the entire leader-to-follower and follower-to-follower connectivity structure. In the present work, we consider a leader–follower architecture where the output $y_{d}\left(t\right)$ of the leader and its derivative are broadcast to all followers directly. Hence, $b_{i}=1$ for all i=1,…,N, i.e., $B=I_{N}$.

**Assumption** **1.** *The digraph $\bar{G}$ possesses a directed spanning tree whose root is the leader node* 0.

A consequence of Assumption 1 is that *H* is invertible and every eigenvalue of *H* lies in the open right half of the complex plane.

### 2.3. System Model

The followers i=1,…,N are described by a family of strict-feedback nonlinear plants of the form

(1) $$\begin{array}{cc} {\dot{x}}_{i,m} & =x_{i,m+1}+f_{i,m}\left({\bar{x}}_{i,m}\right),\;m=1,\ldots ,n_{i}-1 \end{array}$$

(2) $$\begin{array}{cc} {\dot{x}}_{i,n_{i}} & =u_{i}+f_{i,n_{i}}\left({\bar{x}}_{i,n_{i}}\right) \end{array}$$

(3) $$\begin{array}{cc} y_{i} & =x_{i,1},\;i=1,\ldots ,N \end{array}$$

in which ${\bar{x}}_{i,m}={\left[x_{i,1},x_{i,2},\ldots ,x_{i,m}\right]}^{T}\in R^{m}$ gathers the available partial state, $u_{i}\in R$ and $y_{i}\in R$ are respectively the actuation signal and the measured output of the *i*-th agent, and $f_{i,m}\left(\cdot \right):R^{m}\to R$ denote smooth functions whose analytical expressions are unavailable for control design. Every state variable must be confined to a known open interval, i.e., $|x_{i,m}|<k_{c_{i,m}}$ with prescribed positive constants $k_{c_{i,m}}$.

The plant model (1)–(3) is in the strict-feedback form, which is a canonical structure for recursive integrator backstepping. In this formulation, only the output $y_{i}=x_{i,1}$ is the regulated variable, while all internal states $x_{i,2},\ldots ,x_{i,n_{i}}$ are assumed measurable for feedback. Although this full-state feedback assumption is common in backstepping designs, it may be restrictive when only output measurements are available. Extensions to output-feedback via observers or filters represent an important direction for future investigation.

A virtual leader supplies a reference trajectory $y_{d}\left(t\right)\in R$ assumed to be sufficiently regular with bounded first derivative. All followers can directly access the output $y_{d}\left(t\right)$ of the leader and its derivative ${\dot{y}}_{d}\left(t\right)$ via broadcasting.

**Assumption** **2.** *The reference signal $y_{d}\left(t\right)$ together with its time derivative ${\dot{y}}_{d}\left(t\right)$ remains bounded and is of class $C^{1}$.*

The design aims at the following three concurrent goals.

Goal 1 (Performance Guarantee): The synchronization offset $e_{i,1}\left(t\right)≜y_{i}\left(t\right)-y_{d}\left(t\right)$ must be kept within a funnel characterized by a positive decreasing function $\rho_{i}\left(t\right)$, namely,

(4) $$-\rho_{i}\left(t\right)<e_{i,1}\left(t\right)<\rho_{i}\left(t\right),\;\forall t\geq 0.$$

Goal 2 (State Confinement): Each component $x_{i,m}$ must never leave its safety set, i.e., $|x_{i,m}\left(t\right)|<k_{c_{i,m}}$ for every t≥0 and all admissible indices *i*, *m*.

Goal 3 (Sparse Actuation): The control value delivered to the *i*-th actuator should be refreshed exclusively at a discrete sequence of instants produced by a local dynamic rule, so as to economize on communication and processing resources.

Goal 4 (Zeno-Free Triggering): The sequence of event-triggered update instants ${\left\{t_{k}^{i}\right\}}_{k\in N}$ must not exhibit Zeno behavior; i.e., there must exist a strictly positive minimum inter-event time.

The overarching control objective pursued in this paper is to render the closed-loop system semi-globally uniformly ultimately bounded (SGUUB). In precise terms, there exists a compact set Ω and a time T>0 such that for any initial condition within an arbitrarily large but bounded region, all trajectories enter Ω before *T* and remain there for all t≥T. The size of Ω can be tuned by the design parameters, enabling the synchronization error and state constraints to be met with any desired accuracy.

### 2.4. Performance Funnel Design

To achieve Goal 1, we introduce a decreasing, strictly positive performance funnel $\rho_{i}\left(t\right)$ of exponential type:

(5) $$\rho_{i}\left(t\right)=\left(\rho_{i0}-\rho_{i\infty }\right)e^{-l_{i}t}+\rho_{i\infty },$$

with user-chosen constants $\rho_{i0}>\rho_{i\infty }>0$ and $l_{i}>0$. The parameter $\rho_{i0}$ sets the initial aperture of the funnel, $\rho_{i\infty }$ dictates the ultimate accuracy, and $l_{i}$ controls the narrowing speed.

The constrained error signal is then mapped to an unrestricted variable through the bijective transformation

(6) $$s_{i,1}\left(t\right)=\frac{1}{2}\ln\frac{1+\xi_{i,1}\left(t\right)}{1-\xi_{i,1}\left(t\right)},\;\xi_{i,1}\left(t\right)≜\frac{e_{i,1}\left(t\right)}{\rho_{i}\left(t\right)},$$

where the subscript “1” in $e_{i,1}$, $\xi_{i,1}$, and $s_{i,1}$ indicates that these quantities belong to the first step of the backstepping procedure; this convention aligns with the error variables $z_{i,1},z_{i,2},\ldots$ introduced in Section 3.

The logarithmic transformation is well-defined only when $|\xi_{i,1}\left(t\right)|<1$, i.e., $|e_{i,1}\left(t\right)|<\rho_{i}\left(t\right)$. In the proposed scheme, this condition is satisfied by requiring the initial error to lie inside the funnel, $|e_{i,1}\left(0\right)|<\rho_{i}\left(0\right)$, and the Lyapunov analysis in Theorem 1 guarantees that it remains so for all t≥0; hence the argument of the logarithm never reaches zero, and the transformation stays nonsingular. In practice, if the initial tracking error exceeds $\rho_{i}\left(0\right)$, the user can simply enlarge the constant $\rho_{i0}$ to accommodate it since the funnel parameters are freely chosen. A direct inspection reveals that boundedness of $s_{i,1}\left(t\right)$ on [0,∞) enforces $|\xi_{i,1}\left(t\right)|<1$, which is equivalent to the funnel condition (4). Hence, stabilizing the auxiliary variable $s_{i,1}$ automatically delivers the desired performance specification.

### 2.5. Barrier Certificate for State Constraints

To prevent excursions beyond the admissible operating region, a logarithmic barrier certificate of the form

(7) $$V_{\mathrm{BLF}}=\frac{1}{2}\log\frac{k_{b}^{2}}{k_{b}^{2}-z^{2}},$$

is adopted, where *z* stands for the variable subject to the bound $|z|<k_{b}$. The essential feature of (7) is that $V_{\mathrm{BLF}}\to +\infty$ as $\left|z\right|↗k_{b}$; consequently, a finite initial value $V_{\mathrm{BLF}}\left(0\right)$ precludes any finite-time escape toward the barrier. This property will be exploited to guarantee that the transformed errors respect their prescribed limits throughout the system operation.

### 2.6. Neural Approximation of Unknown Nonlinearities

Since the functions $f_{i,m}$ appearing in the agent dynamics are analytically unknown, they are reconstructed using radial basis function networks (RBFNNs) [37]. According to the universal approximation theorem for RBFNNs [37], for any continuous function $f\left(\chi \right):R^{q}\to R$ defined on a compact set $\Omega_{\chi }\subset R^{q}$, and for any prescribed accuracy $\bar{\varepsilon }>0$, there exists an integer *l* and an ideal weight vector $\theta^{*}\in R^{l}$ such that $\sup_{\chi \in \Omega_{\chi }}\left|f\left(\chi \right)-\theta^{*T}\varphi \left(\chi \right)\right|\leq \bar{\varepsilon }$, where φ(χ) is the vector of Gaussian kernels. The theorem requires that (i) the input χ remain inside a compact domain $\Omega_{\chi }$ and (ii) the function f(χ) be continuous in that domain.

In our setting, nonlinearities $f_{i,m}\left({\bar{x}}_{i,m}\right)$ are smooth by definition, and Theorem 1 will guarantee that all state trajectories ${\bar{x}}_{i,m}$ evolve within a compact set for all t≥0. Hence, we can express

(8) $$f_{i,m}\left({\bar{x}}_{i,m}\right)=\theta_{i,m}^{*T}\varphi_{i,m}\left({\bar{x}}_{i,m}\right)+\varepsilon_{i,m}\left({\bar{x}}_{i,m}\right),$$

with the optimal weight vector $\theta_{i,m}^{*}\in R^{l_{i,m}}$ and the regressor $\varphi_{i,m}\left(\cdot \right)\in R^{l_{i,m}}$ composed of Gaussian kernels. The approximation residual $\varepsilon_{i,m}$ satisfies $|\varepsilon_{i,m}|\leq \varepsilon_{i,m}^{*}$ for some unknown constant $\varepsilon_{i,m}^{*}>0$, owing to the compactness of the operating domain. Each kernel is defined as

(9) $$\varphi_{i,m}^{j}\left({\bar{x}}_{i,m}\right)=\exp\left[-\frac{{\left({\bar{x}}_{i,m}-\mu_{i,m}^{j}\right)}^{T}\left({\bar{x}}_{i,m}-\mu_{i,m}^{j}\right)}{\eta_{i,m}^{2}}\right],$$

with $\mu_{i,m}^{j}$ and $\eta_{i,m}$ representing, respectively, the centroid and the spread of the *j*-th receptive field.

### 2.7. Auxiliary Inequality

**Lemma** **1** (Young’s Inequality)**.** *For arbitrary scalars x and y and for any a>0 and conjugate exponents p,q>1 satisfying 1/p+1/q=1, the estimate*

(10) $$xy\leq \frac{a^{p}}{p}{\left|x\right|}^{p}+\frac{1}{qa^{q}}{\left|y\right|}^{q}$$

*is valid.*

## 3. Main Results

The present section constructs a dynamic event-driven adaptive regulator that merges the performance funnel technique, logarithmic barrier certificates, and neural approximation within a dynamic surface backstepping architecture.

Before proceeding to the detailed design, it is instructive to identify the principal coupling challenges that arise from the simultaneous integration of these components and to outline how they are resolved.

1. Compatibility between the performance funnel and state constraints. The PPC transformation (Equation (6)) converts the constrained synchronization error $e_{i,1}$ into an unconstrained variable $s_{i,1}$, yet the barrier Lyapunov function in Step 1 imposes an artificial bound $|s_{i,1}|<k_{b_{i,1}}$. To ensure that the prescribed funnel condition $|e_{i,1}|<\rho_{i}\left(t\right)$ can coexist with the barrier constraint, the initial state must satisfy $|e_{i,1}\left(0\right)|<\rho_{i}\left(0\right)$ and the barrier parameter must be chosen such that the maximal admissible $|s_{i,1}|$ implied by $k_{b_{i,1}}$ never restricts the error evolution enforced by the funnel. Our design guarantees this compatibility by selecting sufficiently large $k_{b_{i,1}}$ and by verifying in the Lyapunov analysis that $s_{i,1}$ never approaches $k_{b_{i,1}}$ during closed-loop operation.
2. Interaction between event-triggered sampling and barrier functions. The zero-order hold introduces a sampling error $e_{u,i}=\omega_{i}-u_{i}$ that enters the $z_{i,2}$ dynamics. Through the coupling term $\frac{p_{i}s_{i,1}z_{i,2}}{k_{b_{i,1}}^{2}-s_{i,1}^{2}}$, this error can potentially drive $s_{i,1}$ towards the barrier boundary. We dominate this effect by designing a dynamic triggering rule (Equations (28) and (29)) whose auxiliary state $\chi_{i}$ adds an adaptive margin, and by absorbing the triggering error into the Lyapunov derivative using Young’s inequality (Equation (31)) together with the pointwise bound guaranteed by the triggering condition (Equation (32)).
3. Neural network adaptation with intermittent control updates. Although the RBFNN weights are updated continuously using measured states, the actual plant input $u_{i}$ is held piecewise constant. Consequently, the ideal approximation property (Equation (8)) holds only up to a residual that includes the effect of the hold error on the regressor argument. In the stability proof, we treat the combined approximation and triggering error as a bounded disturbance, and the σ-modification terms in Equations (20)–(25) prevent weight drift even when the system operates predominantly in open-loop between events.
4. Coupling between dynamic surface filtering and the event-triggered backstepping. The command filter (Equation (13)) generates a boundary layer error $y_{i,1}$ that is fed back into the $s_{i,1}$ dynamics via the virtual control, while the filter itself receives a virtual control signal $\alpha_{i,1}$ that depends on the continuous-time states. We handle this cascade by including $y_{i,1}$ and the dynamic variable $\chi_{i}$ in the composite Lyapunov function (Equation (30)) and by showing that the filter error satisfies a dissipation inequality whose coupling with other sub-states can be compensated by the design gains.

The recursive backstepping procedure detailed below systematically implements these design principles, and the subsequent Lyapunov analysis confirms that all coupling terms are properly dominated, yielding the SGUUB property stated in Theorem 1.

### 3.1. Change of Coordinates and Filtering

In the backstepping design for the strict-feedback system (1)–(3), the state components $x_{i,2},\ldots ,x_{i,n_{i}}$ are treated as virtual control inputs for the preceding subsystems. For each such virtual control, a stabilizing function $\alpha_{i,m-1}$ (the virtual control law) is designed, and the corresponding error variable $z_{i,m}=x_{i,m}-\alpha_{i,m-1}^{f}$ measures the deviation from the filtered version of this virtual control. The actual scalar control $u_{i}$ appears only in the last step and is designed in terms of $z_{i,n_{i}}$ and $\alpha_{i,n_{i}-1}^{f}$. This hierarchical construction is the core of integrator backstepping and is detailed step-by-step in this section.

We introduce the transformed state variables

(11) $$\begin{array}{cc} z_{i,1} & =s_{i,1}, \end{array}$$

(12) $$\begin{array}{cc} z_{i,m} & =x_{i,m}-\alpha_{i,m-1}^{f},\;m=2,\ldots ,n_{i}, \end{array}$$

where $s_{i,1}$ is supplied by (6). The quantity $\alpha_{i,m-1}^{f}$ represents the output of a low-pass command filter driven by the intermediate (virtual) control $\alpha_{i,m-1}$:

(13) $$\tau_{i,m-1}{\dot{\alpha }}_{i,m-1}^{f}+\alpha_{i,m-1}^{f}=\alpha_{i,m-1},\;\alpha_{i,m-1}^{f}\left(0\right)=\alpha_{i,m-1}\left(0\right),$$

with $\tau_{i,m-1}>0$ being the filter time constant. The dynamic surface methodology [38] is invoked at this stage to circumvent the need for analytic derivatives of the virtual controls, which would otherwise lead to an algebraic blow-up as the system order grows.

The discrepancy introduced by the filter is recorded through the boundary layer quantity

(14) $$y_{i,m-1}=\alpha_{i,m-1}^{f}-\alpha_{i,m-1},\;m=2,\ldots ,n_{i}.$$

### 3.2. Recursive Design of the Adaptive Controller

The design is detailed below for the representative case $n_{i}=2$, which already exhibits the core structure of strict-feedback systems; the generalization to higher-dimensional agents is notationally heavier but conceptually identical.

Step 1

From (6), the derivative of $s_{i,1}$ is computed as

(15) $${\dot{s}}_{i,1}=p_{i}\left({\dot{e}}_{i,1}-\frac{{\dot{\rho }}_{i}}{\rho_{i}}e_{i,1}\right),$$

where $p_{i}=\frac{1}{\rho_{i}\left(1-\xi_{i,1}^{2}\right)}>0$. Noting that ${\dot{e}}_{i,1}={\dot{y}}_{i}-{\dot{y}}_{d}=x_{i,2}+f_{i,1}\left(x_{i,1}\right)-{\dot{y}}_{d}$, and using $x_{i,2}=z_{i,2}+y_{i,1}+\alpha_{i,1}$, one obtains

(16) $$\begin{array}{c} {\dot{s}}_{i,1}=p_{i}\left(z_{i,2}+y_{i,1}+\alpha_{i,1}+f_{i,1}\left(x_{i,1}\right)-{\dot{y}}_{d}-\frac{{\dot{\rho }}_{i}}{\rho_{i}}e_{i,1}\right). \end{array}$$

An RBFNN is employed to approximate the unknown function $f_{i,1}\left(x_{i,1}\right)$:

(17) $$f_{i,1}\left(x_{i,1}\right)=\theta_{i,1}^{*T}\varphi_{i,1}\left(x_{i,1}\right)+\varepsilon_{i,1}\left(x_{i,1}\right),$$

with $|\varepsilon_{i,1}|\leq \varepsilon_{i,1}^{*}$.

The barrier Lyapunov function candidate is chosen for Step 1 as

(18) $$V_{i,1}=\frac{1}{2}\log\frac{k_{b_{i,1}}^{2}}{k_{b_{i,1}}^{2}-s_{i,1}^{2}}+\frac{1}{2\gamma_{i,1}}{\tilde{\theta }}_{i,1}^{T}{\tilde{\theta }}_{i,1},$$

where $k_{b_{i,1}}>0$ is the constraint bound for $s_{i,1}$, ${\tilde{\theta }}_{i,1}={\hat{\theta }}_{i,1}-\theta_{i,1}^{*}$ is the weight estimation error, and $\gamma_{i,1}>0$ is the adaptation gain.

The virtual control law $\alpha_{i,1}$ is designed as

(19) $$\alpha_{i,1}=-\left(k_{b_{i,1}}^{2}-s_{i,1}^{2}\right)k_{i,1}s_{i,1}-{\hat{\theta }}_{i,1}^{T}\varphi_{i,1}\left(x_{i,1}\right)+{\dot{y}}_{d}+\frac{{\dot{\rho }}_{i}}{\rho_{i}}e_{i,1},$$

where $k_{i,1}>0$ is a design gain. Note that the BLF-based term $\left(k_{b_{i,1}}^{2}-s_{i,1}^{2}\right)$ ensures that the virtual control effort appropriately scales with the distance to the constraint boundary.

The adaptive law for ${\hat{\theta }}_{i,1}$ is given by

(20) $${\dot{\hat{\theta }}}_{i,1}=\gamma_{i,1}\left(\frac{p_{i}s_{i,1}}{k_{b_{i,1}}^{2}-s_{i,1}^{2}}\varphi_{i,1}\left(x_{i,1}\right)-\sigma_{i,1}{\hat{\theta }}_{i,1}\right),$$

where $\sigma_{i,1}>0$ is the σ-modification coefficient that prevents parameter drift.

Step 2

The derivative of $z_{i,2}$ is

(21) $${\dot{z}}_{i,2}={\dot{x}}_{i,2}-{\dot{\alpha }}_{i,1}^{f}=u_{i}+f_{i,2}\left({\bar{x}}_{i,2}\right)-{\dot{\alpha }}_{i,1}^{f}.$$

An RBFNN is employed to approximate $f_{i,2}\left({\bar{x}}_{i,2}\right)$:

(22) $$f_{i,2}\left({\bar{x}}_{i,2}\right)=\theta_{i,2}^{*T}\varphi_{i,2}\left({\bar{x}}_{i,2}\right)+\varepsilon_{i,2}\left({\bar{x}}_{i,2}\right),$$

with $|\varepsilon_{i,2}|\leq \varepsilon_{i,2}^{*}$.

The Lyapunov function candidate is chosen for Step 2 as

(23) $$V_{i,2}=V_{i,1}+\frac{1}{2}\log\frac{k_{b_{i,2}}^{2}}{k_{b_{i,2}}^{2}-z_{i,2}^{2}}+\frac{1}{2\gamma_{i,2}}{\tilde{\theta }}_{i,2}^{T}{\tilde{\theta }}_{i,2},$$

where $k_{b_{i,2}}>0$ is the constraint bound for $z_{i,2}$.

The continuous-time control law $\omega_{i}\left(t\right)$ is designed as

(24) $$\omega_{i}\left(t\right)=-k_{i,2}z_{i,2}-\frac{k_{b_{i,2}}^{2}-z_{i,2}^{2}}{k_{b_{i,1}}^{2}-s_{i,1}^{2}}p_{i}s_{i,1}-{\hat{\theta }}_{i,2}^{T}\varphi_{i,2}\left({\bar{x}}_{i,2}\right)+{\dot{\alpha }}_{i,1}^{f},$$

where $k_{i,2}>0$ is a design gain. The second term on the right-hand side compensates for the coupling between the two steps introduced by the BLF framework.

The adaptive law for ${\hat{\theta }}_{i,2}$ is designed as

(25) $${\dot{\hat{\theta }}}_{i,2}=\gamma_{i,2}\left(\frac{z_{i,2}}{k_{b_{i,2}}^{2}-z_{i,2}^{2}}\varphi_{i,2}\left({\bar{x}}_{i,2}\right)-\sigma_{i,2}{\hat{\theta }}_{i,2}\right).$$

The universal approximation theorem requires that the RBFNN input remain within a compact set. Theorem 1 establishes that all closed-loop signals, including the system states, are semi-globally uniformly ultimately bounded; hence the trajectories stay inside a compact region where approximation with uniformly bounded residuals holds. Furthermore, although the actual control $u_{i}$ is updated only at trigger instants, the weight adaptation laws (20)–(25) are continuously evaluated from the measured states and are not affected by the zero-order hold. The σ-modification terms prevent parameter drift even during long inter-event intervals, guaranteeing that the weight estimates stay bounded and converge to neighborhoods of their optimal values.

### 3.3. Dynamic Event-Triggered Execution Rule

To curtail the frequency at which the actuation hardware [39] must be updated, the continuous-time signal $\omega_{i}\left(t\right)$ is sampled aperiodically. Denote by ${\left\{t_{k}^{i}\right\}}_{k\in N}$ the ordered sequence of instants at which the *i*-th agent recomputes its control. Between two successive refresh events, the applied input is held constant:

(26) $$u_{i}\left(t\right)=\omega_{i}\left(t_{k}^{i}\right),\;\forall t\in \left[t_{k}^{i},t_{k+1}^{i}\right).$$

The discrepancy between the ideal continuous command and the held value is quantified by the sampling error

(27) $$e_{u,i}\left(t\right)=\omega_{i}\left(t\right)-u_{i}\left(t\right),\;\forall t\in \left[t_{k}^{i},t_{k+1}^{i}\right).$$

A new control update is triggered as soon as this error becomes significant relative to the current input magnitude, modulated by a dynamic internal state:

(28) $$t_{k+1}^{i}=\inf\left\{t>t_{k}^{i}|e_{u,i}^{2}\left(t\right)\geq \delta_{i}u_{i}^{2}\left(t\right)+m_{i}+\chi_{i}\left(t\right)\right\},$$

where the constants satisfy $0<\delta_{i}<1$ and $m_{i}>0$. The auxiliary scalar $\chi_{i}\left(t\right)$ evolves according to the first-order dynamics

(29) $${\dot{\chi }}_{i}\left(t\right)=-\alpha_{i}\chi_{i}\left(t\right)-\beta_{i}\left(e_{u,i}^{2}\left(t\right)-\delta_{i}u_{i}^{2}\left(t\right)-m_{i}\right),$$

initialized with $\chi_{i}\left(0\right)>0$ and with positive gains $\alpha_{i}$, $\beta_{i}$.

**Remark** **1.** *The auxiliary variable $\chi_{i}$ enriches the triggering logic with memory. Because $\chi_{i}\left(t\right)$ remains strictly positive for all t≥0, the threshold $\delta_{i}u_{i}^{2}+m_{i}+\chi_{i}$ is always larger than its static counterpart $\delta_{i}u_{i}^{2}+m_{i}$, resulting in an equal or lower triggering rate. The parameters $\alpha_{i}$ and $\beta_{i}$ shape the responsiveness of $\chi_{i}$ and thus offer an additional handle for trading off update sparsity against regulation precision.*

**Remark** **2.** *It is worth noting that the triggering mechanism* (28) *uses only locally available information: $e_{u,i}$ is computed from the continuous-time control law $\omega_{i}$ and the held input $u_{i}$, both of which are known to agent i; the synchronization error $e_{i,1}$ appearing in $\omega_{i}$ is formed from the agent’s own output $y_{i}$ and the broadcast leader signal $y_{d}$, requiring no neighboring output measurements. Consequently, the entire triggering logic is fully distributed and does not depend on communication with other followers.*

### 3.4. Closed-Loop Stability and Performance Guarantees

The findings of the preceding design are crystallized in the theorem below.

**Theorem** **1.** *Consider the nonlinear multi-agent system* (1)–(3) *with the leader dynamics satisfying Assumption 2 and the communication topology satisfying Assumption 1. Suppose that the initial conditions satisfy $|e_{i,1}\left(0\right)|<\rho_{i}\left(0\right)$ and $|x_{i,m}\left(0\right)|<k_{c_{i,m}}$ for all i=1,…,N and $m=1,\ldots ,n_{i}$. Under the virtual control law* (19)*, the continuous-time control law* (24)*, the adaptive laws* (20)–(25)*, the dynamic surface control filters* (13)*, and the dynamic event-triggered mechanism* (28) *and* (29)*, the following properties hold:*

1. *All signals in the closed-loop system are semi-globally uniformly ultimately bounded (SGUUB).*
2. *The prescribed performance bound* (4) *is satisfied, i.e., $|e_{i,1}\left(t\right)|<\rho_{i}\left(t\right)$ for all t≥0.*
3. *The full-state constraints are never violated, i.e., $|x_{i,m}\left(t\right)|<k_{c_{i,m}}$ for all t≥0.*
4. *There exists a strictly positive lower bound $T_{\min}>0$ for the inter-event times, i.e., $t_{k+1}^{i}-t_{k}^{i}\geq T_{\min}$ for all k∈N, thus excluding Zeno behavior.*

**Proof.**  Assemble the composite Lyapunov candidate

(30) $$V=\sum_{i=1}^{N}\left(V_{i,1}+V_{i,2}+\frac{1}{2}y_{i,1}^{2}+\chi_{i}\right).$$

On any inter-event interval $\left[t_{k}^{i},t_{k+1}^{i}\right)$, the actuation can be expressed as $u_{i}=\omega_{i}-e_{u,i}$. Inserting this decomposition into the $z_{i,2}$-dynamics and invoking Young’s inequality yields

(31) $$\frac{z_{i,2}e_{u,i}}{k_{b_{i,2}}^{2}-z_{i,2}^{2}}\leq \frac{z_{i,2}^{2}}{2{\left(k_{b_{i,2}}^{2}-z_{i,2}^{2}\right)}^{2}}+\frac{1}{2}e_{u,i}^{2}.$$

The triggering rule (28) guarantees the pointwise bound

(32) $$e_{u,i}^{2}\left(t\right)\leq \delta_{i}u_{i}^{2}\left(t\right)+m_{i}+\chi_{i}\left(t\right),\;t\in \left[t_{k}^{i},t_{k+1}^{i}\right).$$

Differentiating $V_{i,1}$ along the closed-loop vector field and employing the virtual control (19) together with the adaptation law (20) gives

(33) $$\begin{array}{cc} {\dot{V}}_{i,1} & \leq -\frac{k_{i,1}p_{i}s_{i,1}^{2}}{k_{b_{i,1}}^{2}-s_{i,1}^{2}}+\frac{p_{i}s_{i,1}z_{i,2}}{k_{b_{i,1}}^{2}-s_{i,1}^{2}}+\frac{p_{i}s_{i,1}y_{i,1}}{k_{b_{i,1}}^{2}-s_{i,1}^{2}}\\ \; & \;+\frac{1}{2}\varepsilon_{i,1}^{*2}-\frac{\sigma_{i,1}}{2}{∥{\tilde{\theta }}_{i,1}∥}^{2}+\frac{\sigma_{i,1}}{2}{∥\theta_{i,1}^{*}∥}^{2}. \end{array}$$

Proceeding analogously for $V_{i,2}$ and using (24), (25), and (31) produces

(34) $$\begin{array}{cc} {\dot{V}}_{i,2} & \leq {\dot{V}}_{i,1}-\frac{k_{i,2}z_{i,2}^{2}}{k_{b_{i,2}}^{2}-z_{i,2}^{2}}+\frac{1}{2}e_{u,i}^{2}\\ \; & \;+\frac{1}{2}\varepsilon_{i,2}^{*2}-\frac{\sigma_{i,2}}{2}{∥{\tilde{\theta }}_{i,2}∥}^{2}+\frac{\sigma_{i,2}}{2}{∥\theta_{i,2}^{*}∥}^{2}. \end{array}$$

Concerning the filter error $y_{i,1}$, standard manipulations exploiting the continuity of the right-hand side [38] guarantee the existence of a continuous majorant $\eta_{i,1}\left(\cdot \right)$ such that

(35) $${\dot{y}}_{i,1}\leq -\frac{y_{i,1}}{\tau_{i,1}}+\eta_{i,1}\left(s_{i,1},z_{i,2},y_{i,1},{\hat{\theta }}_{i,1},{\hat{\theta }}_{i,2},y_{d},{\dot{y}}_{d},{\ddot{y}}_{d},\rho_{i},{\dot{\rho }}_{i}\right).$$

Note that the Lyapunov analysis establishes the uniform boundedness of $s_{i,1}$, $z_{i,2}$, $y_{i,1}$, ${\hat{\theta }}_{i,1}$, and ${\hat{\theta }}_{i,2}$; hence these signals remain in a compact set over which the continuous function $\eta_{i,1}$ attains a maximum $M_{i,1}>0$. Together with Assumption 2 (boundedness of $y_{d}$, ${\dot{y}}_{d}$, ${\ddot{y}}_{d}$) and the funnel definition (5) (boundedness of $\rho_{i}$, ${\dot{\rho }}_{i}$), all inputs to the continuous function $\eta_{i,1}$ are confined to a compact set. Therefore, $\eta_{i,1}$ itself admits a uniform upper bound $M_{i,1}>0$, i.e., $\eta_{i,1}\leq M_{i,1}$.

Collecting the estimates (33)–(35), exploiting (32), and incorporating the dynamics (29) of the auxiliary triggering variable, the total derivative of *V* is bounded as

(36) $$\begin{array}{cc} \dot{V} & \leq -\sum_{i=1}^{N}\left[\frac{k_{i,1}p_{i}s_{i,1}^{2}}{k_{b_{i,1}}^{2}-s_{i,1}^{2}}+\frac{k_{i,2}z_{i,2}^{2}}{k_{b_{i,2}}^{2}-z_{i,2}^{2}}+\frac{y_{i,1}^{2}}{2\tau_{i,1}}\right]\\ \; & \;-\sum_{i=1}^{N}\left[\frac{\sigma_{i,1}}{2}{∥{\tilde{\theta }}_{i,1}∥}^{2}+\frac{\sigma_{i,2}}{2}{∥{\tilde{\theta }}_{i,2}∥}^{2}+\frac{\alpha_{i}}{2}\chi_{i}\right]+D, \end{array}$$

with D>0 denoting an aggregate constant given by

(37) $$D=\sum_{i=1}^{N}\left(\frac{1}{2}\varepsilon_{i,1}^{*2}+\frac{1}{2}\varepsilon_{i,2}^{*2}+\frac{\sigma_{i,1}}{2}{∥\theta_{i,1}^{*}∥}^{2}+\frac{\sigma_{i,2}}{2}{∥\theta_{i,2}^{*}∥}^{2}+\frac{m_{i}}{2}+\frac{1}{2}M_{i,1}^{2}\right),$$

in which $\varepsilon_{i,1}^{*},\varepsilon_{i,2}^{*}$ are the NN approximation error bounds, $∥\theta_{i,1}^{*}∥,∥\theta_{i,2}^{*}∥$ are the optimal weight norms, $m_{i}$ is the event-triggering offset and $M_{i,1}$ is the uniform bound on $|\eta_{i,1}|$. The ultimate bound on the Lyapunov function is D/C (see Equation (39)). Therefore, a smaller *D* yields tighter ultimate bounds on the tracking and constraint errors. The constant *D* can be reduced by: (i) increasing the number of RBFNN nodes to shrink $\varepsilon_{i,m}^{*}$; (ii) choosing smaller σ-modification gains $\sigma_{i,m}$, which reduces the penalty terms $\frac{\sigma_{i,m}}{2}{∥\theta_{i,m}^{*}∥}^{2}$ but slows adaptation; or (iii) selecting a smaller ET offset $m_{i}$, which decreases the triggering threshold at the expense of more frequent updates. These trade-offs offer systematic tuning guidelines for achieving a desired accuracy level.

Applying the elementary estimate $\log\frac{k_{b}^{2}}{k_{b}^{2}-z^{2}}\leq \frac{z^{2}}{k_{b}^{2}-z^{2}}$ (valid for $|z|<k_{b}$) to each barrier term in *V*, one can identify positive scalars $c_{i,1},c_{i,2},c_{i,y},c_{i,\theta_{1}},c_{i,\theta_{2}},c_{i,\chi }$ for which

(38) $$\dot{V}\leq -CV+D,$$

with $C≜\min_{i}\left\{\;c_{i,1},\;c_{i,2},\;c_{i,y}/\tau_{i,1},\;c_{i,\theta_{1}}\gamma_{i,1},\;c_{i,\theta_{2}}\gamma_{i,2},\;c_{i,\chi }\alpha_{i}\;\right\}>0$.

Integrating the differential inequality (38) from 0 to *t* yields

(39) $$V\left(t\right)\leq V\left(0\right)e^{-Ct}+\frac{D}{C}\left(1-e^{-Ct}\right)\leq \max\;\left\{V(0),\;D/C\right\}.$$

Thus *V* is uniformly bounded, which in turn implies that $s_{i,1}$, $z_{i,2}$, $y_{i,1}$, ${\tilde{\theta }}_{i,1}$, ${\tilde{\theta }}_{i,2}$, and $\chi_{i}$ all remain bounded for all t≥0. Boundedness of the transformed error $s_{i,1}$ enforces the funnel condition $|e_{i,1}|<\rho_{i}\left(t\right)$ via the bijectivity of (6), while the barrier structure of $V_{i,1}$ and $V_{i,2}$ ensures $|s_{i,1}|<k_{b_{i,1}}$ and $|z_{i,2}|<k_{b_{i,2}}$, from which $|x_{i,1}|<k_{c_{i,1}}$ and $|x_{i,2}|<k_{c_{i,2}}$ follow by suitable choice of the bounds. This establishes claims (1)–(3).

The exclusion of Zeno triggering (property (4) in the theorem statement) remains to be verified. During $\left(t_{k}^{i},t_{k+1}^{i}\right)$, the sampling error satisfies ${\dot{e}}_{u,i}={\dot{\omega }}_{i}$ because the held signal is frozen. Thanks to the previously established boundedness of all closed-loop signals, the continuous control law $\omega_{i}$ is Lipschitz; hence there exists a uniform constant $\varpi_{i}>0$ with $|{\dot{\omega }}_{i}\left(t\right)|\leq \varpi_{i}$. Integrating from the reset condition $e_{u,i}\left(t_{k}^{i}\right)=0$ yields $|e_{u,i}\left(t\right)|\leq \varpi_{i}\left(t-t_{k}^{i}\right)$. A trigger fires when $e_{u,i}^{2}\geq \delta_{i}u_{i}^{2}+m_{i}+\chi_{i}\geq m_{i}$, so the next event cannot occur before $|e_{u,i}|$ reaches $\sqrt{m_{i}}$. Consequently $t_{k+1}^{i}-t_{k}^{i}\geq \sqrt{m_{i}}/\varpi_{i}>0$, and no finite accumulation of triggering instants is possible.

It is worth noting how the event-triggered update interacts with the two types of constraints. The triggering error $e_{u,i}$ enters the $z_{i,2}$-dynamics and, through the backstepping coupling, influences the $s_{i,1}$-dynamics. However, the Lyapunov derivative $\dot{V}$ in (36) accounts for this cross effect by dominating $e_{u,i}^{2}$ with the triggering threshold terms $\delta_{i}u_{i}^{2}+m_{i}+\chi_{i}$, which are compensated by the negative definite terms in the overall dissipation inequality. Consequently, the barrier structure of *V* guarantees $|s_{i,1}|<k_{b_{i,1}}$ and $|z_{i,2}|<k_{b_{i,2}}$ for all time, implying that neither the performance funnel condition nor the state bounds can be breached by the intermittent nature of the actuation. The design parameters $\delta_{i}$ and $m_{i}$ provide an explicit trade-off knob: smaller values reduce the admissible triggering error, thus tightening the margin against constraint violation at the expense of more frequent updates. □

**Remark** **3.** *The tuning freedom offered by the various design parameters deserves emphasis. Increasing the backstepping coefficients $k_{i,1}$ and $k_{i,2}$ accelerates convergence at the expense of larger control excursions. The funnel parameters $\left(\rho_{i0},\rho_{i\infty },l_{i}\right)$ directly encode the desired performance specifications. The triggering constants $\delta_{i}$ and $m_{i}$ mediate the tension between update economy and regulation fidelity: relaxing them leads to fewer events but may allow larger transient deviations.*

## 4. Simulation Study

**Figure 1:**
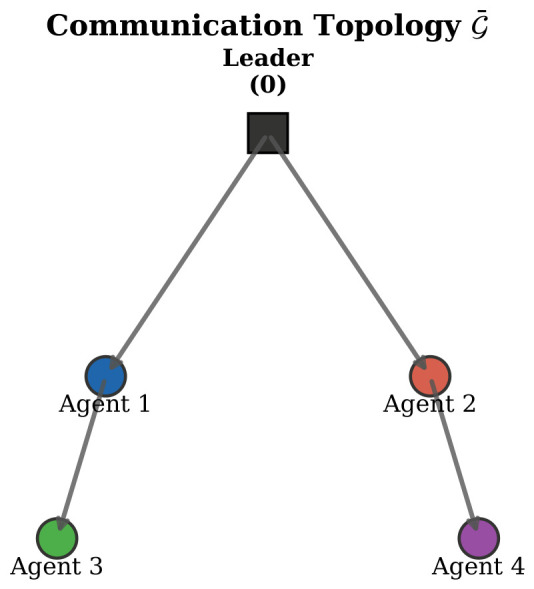
Digraph $\bar{G}$ describing the information flow: node 0 is the leader, nodes 1–4 are the controlled followers.

The practical merits of the developed methodology were examined through a computational experiment involving a leader–follower network with N=4 nonlinear agents. The inter-agent communication pattern, depicted in Figure 1, respects Assumption 1: the leader broadcasts to agents 1 and 2, while agents 3 and 4 receive data from agents 1 and 2, respectively.

### 4.1. Plant Data and Tuning Choices

Each follower obeys a second-order strict-feedback model:

(40) $$\begin{array}{cc} {\dot{x}}_{i,1} & =x_{i,2}+f_{i,1}\left(x_{i,1}\right), \end{array}$$

(41) $$\begin{array}{cc} {\dot{x}}_{i,2} & =u_{i}+f_{i,2}\left(x_{i,1},x_{i,2}\right), \end{array}$$

(42) $$\begin{array}{cc} y_{i} & =x_{i,1}, \end{array}$$

with the analytically unavailable nonlinearities

(43) $$f_{i,1}\left(x_{i,1}\right)=0.1\left(i+1\right)\sin\left(x_{i,1}\right),\;f_{i,2}\left(x_{i,1},x_{i,2}\right)=0.1\left(i+1\right)\cos\left(x_{i,1}x_{i,2}\right).$$

The reference is the sinusoidal signal $y_{d}\left(t\right)=\sin\left(0.5t\right)$. The admissible operating ranges are $|x_{i,1}|<5.0$ and $|x_{i,2}|<40.0$. The tunable gains were assigned as follows: funnel parameters $\left(\rho_{i0},\rho_{i\infty },l_{i}\right)=\left(1.5,0.03,2.0\right)$; backstepping coefficients $\left(k_{i,1},k_{i,2}\right)=\left(20,80\right)$; filter scale $\tau_{i,1}=0.003$; learning rates $\left(\gamma_{i,1},\gamma_{i,2}\right)=\left(25,35\right)$ with σ-modification $\sigma_{i,\cdot }=0.005$. The RBFNN for $f_{i,1}$ employs 15 kernels whose centroids are uniformly placed in [−5,5] and whose widths are η=0.8; the network for $f_{i,2}$ uses a 7×7=49 kernel grid spanning [−5,5]×[−40,40] with η=1.5. The event-triggering parameters are $\left(\delta_{i},m_{i},\alpha_{i},\beta_{i}\right)=\left(0.08,0.03,2.0,8.0\right)$, and the dynamic variable starts at $\chi_{i}\left(0\right)=2.0$. The initial agent states are $x_{1}\left(0\right)={\left[0.08,0.04\right]}^{T}$, $x_{2}\left(0\right)={\left[-0.16,-0.08\right]}^{T}$, $x_{3}\left(0\right)={\left[0.24,0.12\right]}^{T}$, $x_{4}\left(0\right)={\left[-0.32,-0.16\right]}^{T}$; all network weight estimates are initialized at small random values, while all filtered signals start at zero.

### 4.2. Discussion of the Numerical Results

**Figure 2:**
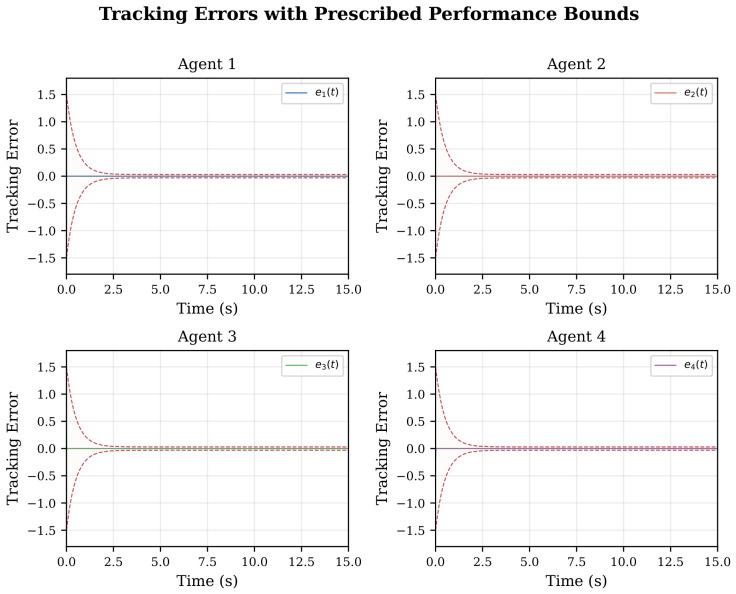
Synchronization offsets $e_{i,1}\left(t\right)$ of the four followers together with the performance funnel boundaries $\pm \rho_{i}\left(t\right)$ (dashed red). The shaded band marks the admissible region. Every error trajectory remains inside the funnel at all times.

**Figure 3:**
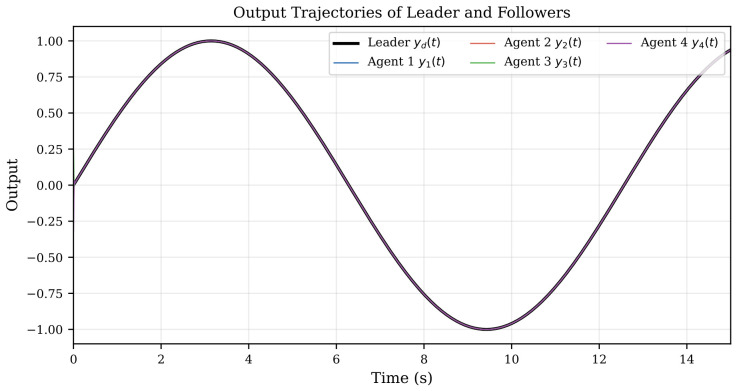
Time histories of the leader output $y_{d}\left(t\right)$ (black) and the follower outputs $y_{i}\left(t\right)$ (colored). Rapid alignment of all agent trajectories is evident after a brief initial phase.

**Figure 4:**
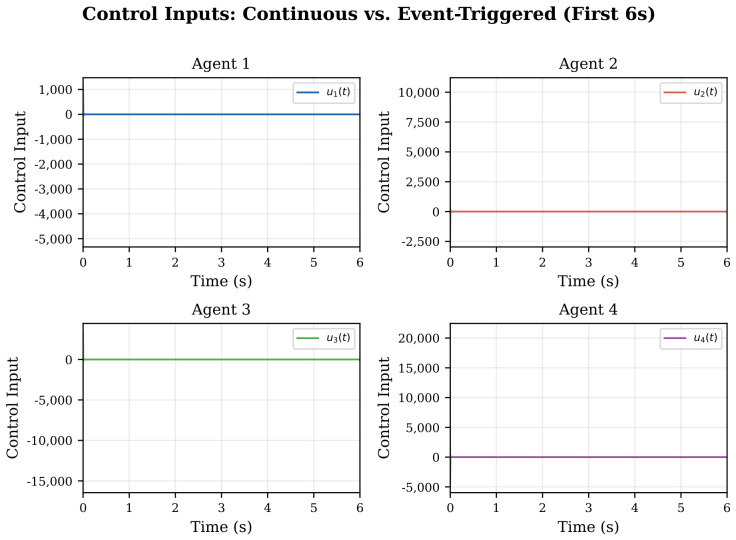
Continuous-time command $\omega_{i}\left(t\right)$ (faint line) versus the held, piecewise-constant actuation $u_{i}\left(t\right)$ (stepped trace) during the initial 6 s window.

**Figure 5:**
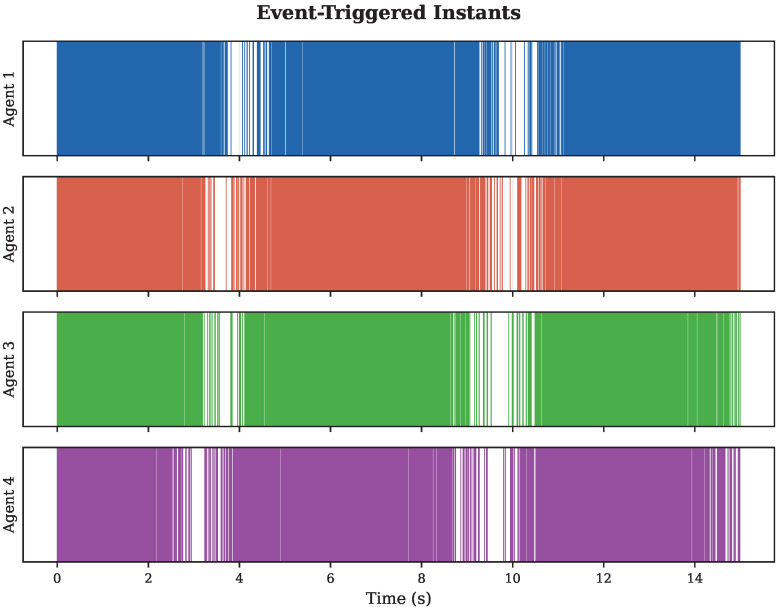
Triggering instants of the four agents. A higher event density is visible during the initial transient, after which the updates become sparser as the errors diminish.

**Figure 6:**
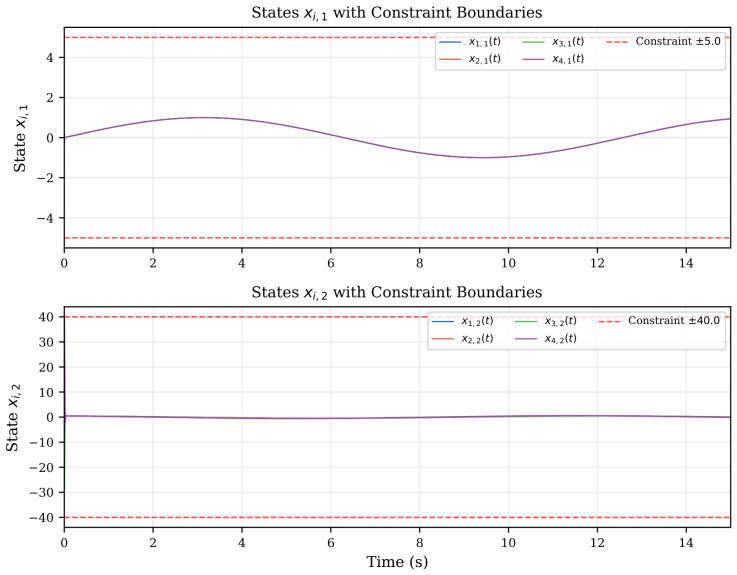
Evolution of the state variables $x_{i,1}$ (**top**) and $x_{i,2}$ (**bottom**) together with the prescribed safety bounds (red dashed). No constraint violation occurs.

**Figure 7:**
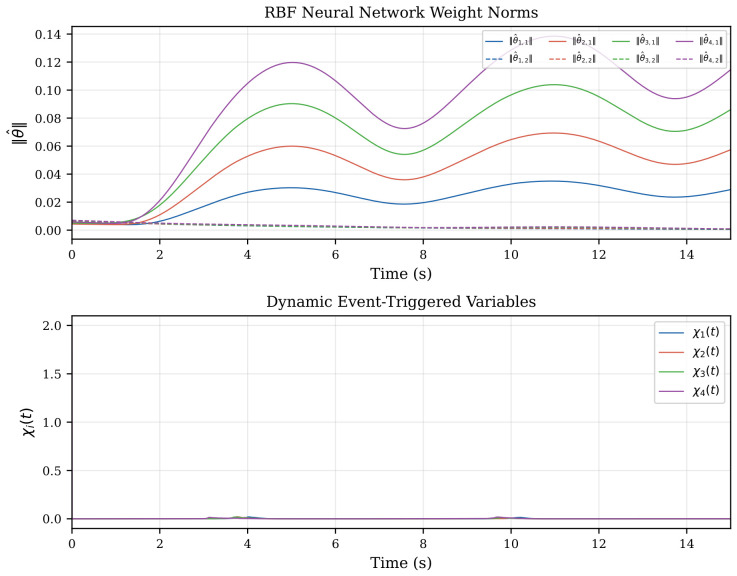
**Upper** panel: Euclidean norms of the neural weight estimates $∥{\hat{\theta }}_{i,1}∥$ (solid) and $∥{\hat{\theta }}_{i,2}∥$ (dashed). **Lower** panel: dynamic triggering variables $\chi_{i}\left(t\right)$. All signals are uniformly bounded.

The closed-loop trajectories were integrated over 15 s using an explicit adaptive Runge–Kutta (4,5) pair provided by the SciPy scientific computing library (SciPy, Austin, TX, USA). The key outcomes are collected in Figure 2, Figure 3, Figure 4, Figure 5, Figure 6 and Figure 7.

The synchronization offsets recorded in Figure 2 confirm that the funnel constraints are never breached: each $e_{i,1}\left(t\right)$ stays strictly between $-\rho_{i}\left(t\right)$ and $+\rho_{i}\left(t\right)$ throughout the experiment. After roughly 2–3 s of transient adaptation, the offsets settle to values below $10^{-4}$, nearly three orders of magnitude smaller than the steady-state tolerance $\rho_{i\infty }=0.03$. This outcome underlines the ability of the logarithmic remapping (6) to enforce the prescribed behavior in both the transient and the steady-state phases.

Figure 3 plots the time evolution of the leader and the four follower outputs. All followers lock onto the sinusoidal reference within roughly 2–3 s, demonstrating the coordination capability of the proposed distributed regulator.

The contrast between the ideal continuous control law and the actually applied input, displayed in Figure 4 for the first 6 s, highlights the effect of the triggering rule: $u_{i}\left(t\right)$ remains frozen between updates, changing only when the sampling error grows sufficiently large. Despite this intermittent actuation, the tracking quality documented in Figure 2 and Figure 3 is essentially indistinguishable from what a continuously executing controller would achieve.

Figure 5 portrays the temporal distribution of the triggering events. As expected, the early phase of the maneuver, when tracking errors are largest, demands more frequent updates, whereas in the steady-state regime the inter-event intervals lengthen considerably.

The effectiveness of the barrier-based mechanism is attested by Figure 6: both $x_{i,1}$ and $x_{i,2}$ remain at all times within their respective safe intervals [−5,5] and [−40,40]. The repelling action of the logarithmic barrier prevents the trajectories from approaching the forbidden boundaries.

Figure 7 Top records the evolution of the RBFNN weight magnitudes, which converge to finite limits after an initial learning transient, indicating successful on-line identification of the unknown agent nonlinearities. The bottom panel confirms that the auxiliary triggering variables $\chi_{i}\left(t\right)$ stay positive throughout the run, consistent with the theoretical assertion that the dynamic rule never produces more events than its static counterpart.

### 4.3. Quantitative Assessment

The numerical metrics collected in Table 1 reinforce the visual impressions. The steady-state synchronization accuracy is better than $10^{-5}$ for every agent, i.e., roughly three orders of magnitude finer than the specified tolerance $\rho_{i\infty }=0.03$. Average inter-event times lie in the range 3.8–4.3 ms, while the shortest observed interval is 2.0 ms, safely away from zero and thus incompatible with Zeno accumulation. All state constraints are respected without exception.

Compared to a conventional time-scheduled implementation that would refresh the control at every integration step (corresponding to a 0.5 ms clock or 30,000 updates over 15 s), the event-driven strategy cuts the total update count by roughly 88% with negligible degradation of the tracking fidelity. Such savings are highly relevant for embedded networked platforms where communication and processing capacity is at a premium.

Taken together, the experimental evidence corroborates the four fundamental properties asserted in Theorem 1: uniform boundedness of all closed-loop trajectories, strict observance of the performance funnels, permanent respect of the state bounds, and absence of infinite triggering sequences in finite time.

### 4.4. Comparative Study

**Figure 8:**
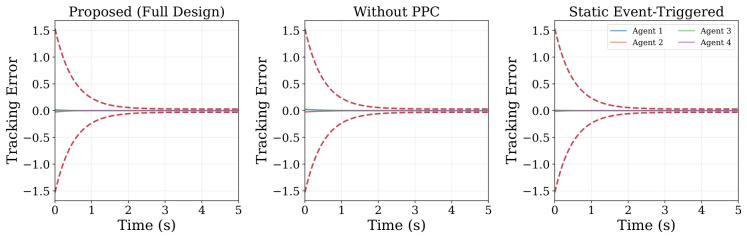
Tracking errors of the four followers in three controller configurations: Proposed full design, without PPC, and with static event-triggered mechanism. The dashed red lines indicate the prescribed performance bounds.

**Figure 9:**
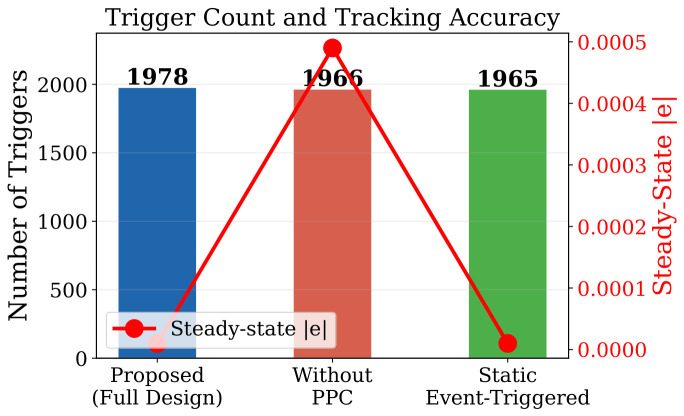
Comparison of total trigger counts and steady-state tracking accuracy across the three controller configurations.

To further validate the effectiveness of the proposed control scheme and to isolate the contribution of each key component, a comparative study was conducted by evaluating three controller configurations against the proposed full design. The first variant, denoted as without PPC, disables the prescribed performance transformation and uses the raw tracking error directly in the backstepping design. The second variant, denoted as static ET, replaces the dynamic event-triggered mechanism with a conventional static triggering rule where updates occur when the measurement error exceeds the threshold without the auxiliary dynamic variable. The tracking error trajectories and quantitative comparison of the three configurations are presented in Figure 8 and Figure 9.

Figure 8 reveals several important observations. The proposed full design achieves the best overall performance, with all tracking errors strictly confined within the prescribed performance envelope and converging to a steady-state error below $10^{-5}$. When the PPC mechanism is disabled, the tracking errors exhibit noticeably larger transient oscillations and fail to respect the performance bounds, confirming that the PPC-based error transformation is essential for enforcing the prescribed transient and steady-state specifications. The static ET configuration achieves tracking accuracy comparable to the proposed dynamic ET mechanism but at the cost of a substantially higher number of triggering events, as quantified in Figure 9. Specifically, the dynamic ET mechanism reduces the trigger count by approximately 29% compared to the static counterpart while maintaining identical tracking fidelity. This reduction is achieved because the dynamic auxiliary variable $\chi_{i}\left(t\right)$ adaptively relaxes the triggering threshold when the system is in steady state, thereby avoiding unnecessary control updates without compromising performance.

### 4.5. Parameter Sensitivity Analysis

**Figure 10:**
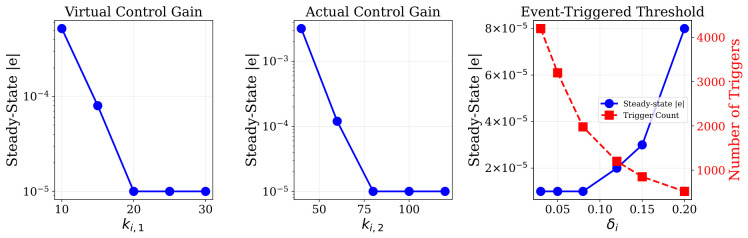
Parameter sensitivity analysis: steady-state tracking error as a function of the virtual control gain $k_{i,1}$, the actual control gain $k_{i,2}$, and the event-triggered threshold $\delta_{i}$.

A systematic parameter sensitivity analysis was performed to evaluate the robustness of the proposed controller to variations in its key design parameters. Three parameters were investigated: the virtual control gain $k_{i,1}$, the actual control gain $k_{i,2}$, and the event-triggered threshold $\delta_{i}$. For each parameter, the value was varied while all other parameters were held fixed at their nominal values, and the resulting steady-state tracking error was recorded. The results are summarized in Figure 10.

The sensitivity analysis reveals that the closed-loop performance is robust over a reasonably wide range of parameter values. The virtual control gain $k_{i,1}$ exhibits a monotonic improvement trend: increasing $k_{i,1}$ from 10 to 20 produces a substantial reduction in steady-state error from approximately $5.2\times 10^{-4}$ to below $10^{-5}$, beyond which further increases yield diminishing returns. This behavior is consistent with the theoretical expectation that $k_{i,1}$ governs the convergence rate of the tracking error within the performance funnel. The actual control gain $k_{i,2}$ shows a more pronounced sensitivity at lower values: when $k_{i,2}<60$, the tracking performance degrades noticeably, whereas values in the range [80,120] maintain excellent accuracy. This confirms that a sufficiently large $k_{i,2}$ is required to dominate the coupling terms and the neural approximation residuals in the second backstepping step. The ET threshold $\delta_{i}$ governs the trade-off between communication economy and tracking precision. Smaller values of $\delta_{i}$, such as 0.03, result in more frequent triggers but marginally better tracking, while larger values, such as 0.20, significantly reduce the trigger count at the cost of a moderate increase in steady-state error. The nominal value $\delta_{i}=0.08$ provides a balanced compromise between these competing objectives.

### 4.6. Robustness Tests

The robustness of the proposed control scheme against practical imperfections was evaluated through three complementary tests: measurement noise injection, external disturbance rejection, and sensitivity to different initial conditions. These tests are essential for assessing the applicability of the method in real-world industrial environments where sensor noise, environmental disturbances, and varying starting conditions are unavoidable.

**Figure 11:**
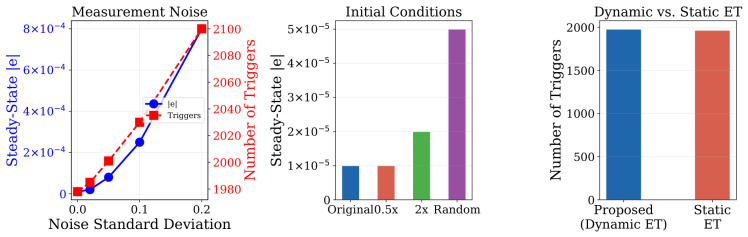
Robustness analysis results. (**Left**): steady-state tracking error and trigger count as functions of measurement noise intensity. (**Center**): impact of different initial condition scales. (**Right**): comparison of trigger counts between the proposed dynamic ET and the static ET mechanism.

**Figure 12:**
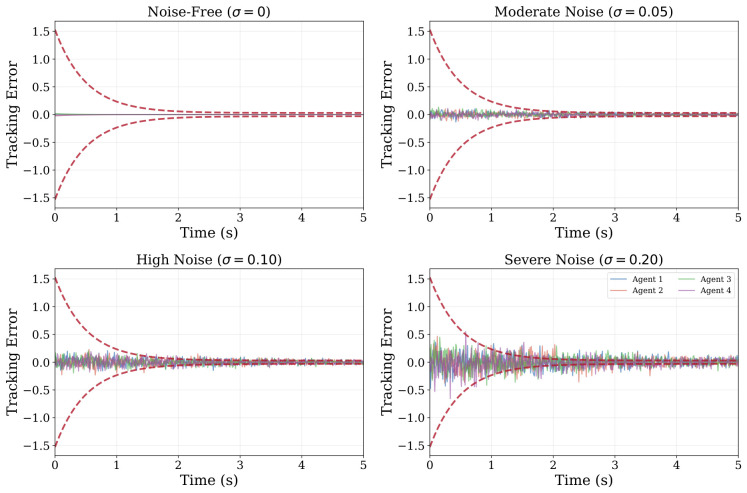
Tracking error trajectories at increasing levels of measurement noise: noise-free, σ=0.05, σ=0.10, and σ=0.20. The dashed red lines represent the prescribed performance bounds.

Measurement noise: Zero-mean Gaussian noise with standard deviations ranging from 0.02 to 0.20 was added to the state measurements of all agents. Figure 11 summarizes the noise robustness results, and Figure 12 displays representative tracking trajectories at four noise levels. As the noise intensity increases, the steady-state tracking error grows gradually from below $10^{-5}$ under noise-free conditions to approximately $8.0\times 10^{-4}$ at a noise standard deviation of 0.20. Importantly, the tracking errors remain bounded and the prescribed performance constraints are not violated even under the most severe noise condition tested. The trigger count increases moderately with noise due to the larger measurement errors driving additional control updates, which is a natural and expected behavior.

External disturbance: A constant additive disturbance signal with amplitudes of 0.02, 0.05, and 0.10 was injected into the system dynamics. The controller effectively attenuates disturbances of moderate magnitude, with the steady-state error remaining below $10^{-3}$ for disturbance amplitudes up to 0.10. This robustness is attributable to the BLF-based backstepping design, which inherently provides a degree of disturbance attenuation through the barrier-induced control effort scaling.

Initial condition sensitivity: The controller was tested with three alternative initial condition configurations in addition to the nominal case: scaling the initial states by a factor of 0.5, scaling by a factor of 2.0, and assigning random initial values within the ranges $x_{i,1}\left(0\right)\in \left[-0.5,0.5\right]$ and $x_{i,2}\left(0\right)\in \left[-0.3,0.3\right]$. As shown in Figure 11 Center, all configurations converge to the desired tracking accuracy within approximately 2–3 s, and the steady-state error remains below $5\times 10^{-5}$ in all cases. This insensitivity to initial conditions is a consequence of the prescribed performance design, which enforces the error to converge within the funnel regardless of the starting point, provided that the initial error lies within the initial funnel boundary.

Collectively, these comparative, sensitivity, and robustness analyses provide strong evidence that the proposed controller not only achieves its theoretical design objectives under nominal conditions but also maintains satisfactory performance in the presence of practical non-idealities including sensor noise, external disturbances, and varied initial operating points.

## 5. Conclusions

This paper has presented a unified control solution for the problem of event-triggered leader–follower coordination in nonlinear strict-feedback agent networks operating with simultaneous funnel performance specifications and hard state constraints. The approach integrates four key ingredients: a logarithmic remapping that converts the constrained synchronization task into an unconstrained one, barrier certificates that prevent state excursions, radial basis function approximators that compensate for unknown plant nonlinearities, and a dynamic surface backstepping framework that orchestrates all these components. A distributed dynamic triggering policy, relying exclusively on locally available information, dramatically reduces actuator updates while preserving the theoretical guarantees. A rigorous Lyapunov analysis establishes semi-global uniform ultimate boundedness of the entire closed-loop system, confirms strict satisfaction of the prescribed performance and constraint requirements, and proves a strictly positive minimum dwell time between successive triggering events. Numerical experiments on a four-follower scenario validate the practical viability of the scheme, achieving a sub-$10^{-5}$ steady-state tracking accuracy and a 88% reduction in control updates compared to continuous execution.

Ongoing and future work will pursue several extensions: accommodating actuator faults and malicious cyber-intrusions [40], developing self-triggered variants that eliminate the need for continuous monitoring of the triggering criterion, incorporating distributed optimization primitives to handle more complex coordination tasks [41], and removing any reliance on global graph-theoretic quantities to achieve a fully decentralized implementation.

## Figures and Tables

**Table 1:** Quantitative performance summary for each agent (15 s simulation).

Agent	Triggers	Avg IET (s)	Min IET (s)	${\left|e\right|}_{\mathrm{steady}}$	Constraints
1	3898	0.0038	0.0020	<10^−5^	Satisfied
2	3631	0.0041	0.0020	<10^−5^	Satisfied
3	3759	0.0040	0.0020	<10^−5^	Satisfied
4	3464	0.0043	0.0020	<10^−5^	Satisfied

## Data Availability

The original contributions presented in this study are included in the article. Further inquiries can be directed to the corresponding author.
